# How do public health professionals view and engage with research? A qualitative interview study and stakeholder workshop engaging public health professionals and researchers

**DOI:** 10.1186/s12889-017-4896-1

**Published:** 2017-11-22

**Authors:** Peter van der Graaf, Lynne F. Forrest, Jean Adams, Janet Shucksmith, Martin White

**Affiliations:** 1 School of Health and Social Care, Teesside University, Middlesbrough, UK; 2 Administrative Data Research Centre Scotland, University of Edinburgh, Edinburgh, UK; 3 MRC Epidemiology Unit, University of Cambridge, Cambridge, UK

**Keywords:** (MESH): Decision making, Public health, Qualitative research, Research personnel, Translational medical research

## Abstract

**Background:**

With increasing financial pressures on public health in England, the need for evidence of high relevance to policy is now stronger than ever. However, the ways in which public health professionals (PHPs) and researchers relate to one another are not necessarily conducive to effective knowledge translation. This study explores the perspectives of PHPs and researchers when interacting, with a view to identifying barriers to and opportunities for developing practice that is effectively informed by research.

**Methods:**

This research focused on examples from two responsive research schemes, which provide university-based support for research-related enquiries from PHPs: the NIHR SPHR Public Health Practitioner Evaluation Scheme^1^ and the responsive research service AskFuse^2^. We examined enquiries that were submitted to both between 2013 and 2015, and purposively selected eight enquiries for further investigation by interviewing the PHPs and researchers involved in these requests. We also identified individuals who were eligible to make requests to the schemes but chose not to do so. In-depth interviews were conducted with six people in relation to the PHPES scheme, and 12 in relation to AskFuse. The interviews were transcribed and analysed using thematic framework analysis. Verification and extension of the findings were sought in a stakeholder workshop.

**Results:**

PHPs recognised the importance of research findings for informing their practice. However, they identified three main barriers when trying to engage with researchers: 1) differences in timescales; 2) limited budgets; and 3) difficulties in identifying appropriate researchers. The two responsive schemes addressed some of these barriers, particularly finding the right researchers to work with and securing funding for local evaluations. The schemes also supported the development of new types of evidence. However, other barriers remained, such as differences in timescales and the resources needed to scale-up research.

**Conclusions:**

An increased mutual awareness of the structures and challenges under which PHPs and researchers work is required. Opportunities for frequent and meaningful engagement between PHPs and researchers can help to overcome additional barriers to co-production of evidence. Collaborative models, such as the use of researchers embedded in practice might facilitate this; however, flexible research funding schemes are needed to support these models.

## Background

Evidence-based policy making is predicated on the value of making decisions about interventions and services on the best available research findings [1]. Public health researchers increasingly seek to produce research of relevance to public health professionals (PHPs) [2]. The need for closer interaction between those working in public health policy and practice, which are referred to here as PHPs, and researchers has long been recognised [3]. In England, this interaction has changed since the responsibility for public health delivery moved in 2013 from National Health Service to local government, which have different approaches to evidence use [4, 5]. Moreover, increasing financial pressures in local government (including public health departments) are driving a need to spend wisely on what works to improve health. This pressure is not unique to the UK and is currently felt across the globe [6].

However, the ways that PHPs can effectively relate to, interact with, undertake and commission research from university researchers to support the development of evidence-based practice are not clear. Previous research, both in the UK and internationally, suggests that difficulties for collaborative research are threefold: PHPs do not know how to access research findings [7]; research timescales often do not align with the policy process [2]; and policy makers and researchers tend to value different types of evidence [8].

The difficulties for collaborative research suggest a need for opportunities for researchers and PHPs to work together to generate research findings of greater utility to public health practice. Several research organisations in England have experimented with new services and programmes to create such opportunities. Two examples are the Public Health Practitioner Evaluation Scheme (PHPES) run by the National Institute for Health Research School for Public Health Research (NIHR SPHR); and the “AskFuse” service, established by Fuse, UKCRC Centre for Translational Research in Public Health.

PHPES [9] is a national, competitive scheme that offers PHPs support to evaluate local interventions in collaboration with SPHR researchers. The scheme was introduced in 2013 by SPHR to give access to researchers in its member organisations, which comprise eight leading public health research centres in England. PHPES aims to produce high quality evidence needed by PHPs to improve population health and reduce health inequalities. PHPs can apply to the scheme for SPHR members to evaluate their local public health interventions. The scheme particularly focuses local, rather than national, public health initiatives that have not been the subject of previous robust evaluations, but which have potential to be applicable elsewhere and have secured operational funding for the research period.

AskFuse [10] is a responsive research facility, based in the north east of England. Introduced in 2013 by Fuse, AskFuse invites local PHPs to approach the service with problems that they think need researching. As such, AskFuse fulfils a broader remit than the PHPES scheme. AskFuse helps PHPs define their research questions and identify the best approach to answering them, and brokers relationships with suitable researchers. Applicants to both PHPES and AskFuse who are not successful in obtaining support are offered advice and referral to other organisations and funding sources, such as regional Research Design Services.

Both schemes aim to increase access to research for PHPs and reduce the time it takes to initiate and carry out research to influence practice and decision making. They increase access and reduce time by enabling early conversations between researchers and PHPs about the most valuable and feasible evidence to answer the questions they ask.

Our study sought to answer the following questions: do response research schemes help PHPs and researchers to overcome the known barriers to collaboration? Does use of the schemes raises new questions and issues? The research was not an evaluation of the response research services; we were more generally interested in the barriers and facilitators to collaboration between PHPs and researchers identified by those involved in these services.

## Methods

### Sampling

We examined research requests that had been submitted to PHPES and AskFuse and identified requests between 2013 and 2015for in-depth, qualitative interviews. We included both instances that did and did not result in successful co-working in each scheme. Twelve enquiries that had resulted in co-working were purposively selected (Eight supported enquiries from AskFuse and four from PHPES that had been successful in receiving funding) to reflect both the range of request types submitted and the range of organisations submitting them. We also sought information on eight requests (four from each scheme) where successful co-working had not been achieved. In total, we sampled 20 cases (12 from AskFuse and eight from PHPES). The sample size was subject to pragmatic, resource-based limitations. Our final sample is described in Table 1.

**Table 1:** Sample characteristics

Service	Public health professional (by sector where relevant)			Supporting researchers		Non appliers/ not funded	
		Invited	Interviewed	Invited	Interviewed	Invited^1^	Interviewed
NIHR SPHR PHPES	LA - public health	3	2	4	3	4	1
		Invited	Interviewed	Invited	Interviewed	Invited^2^	Interviewed
AskFuse projects	NHS LA public health LA non-public health Other	1 4 2 1	1 2 1 1	5	4	4	3
Total		11	7	9	7	8	4

1 PHPs who have applied for NIHR SPHR PHPES support but have not been awarded funding

2 PHPs who are eligible to access AskFuse but have not contacted the service for support

### Inclusion criteria

Since PHPES is a competitive scheme, we selected four applications for inclusion, which had been submitted to the scheme but had not received funding. In relation to AskFuse, which does not have a competitive element, we simply asked PHPs who did use the service to suggest colleagues who were eligible for support from AskFuse but had never contacted the service. With AskFuse we attempted to look for examples of co-operation across a range of enquiry types (request for research digest, support with intervention development, rapid evaluation of services and full evaluation), but this was less relevant for the PHPES scheme where the focus is almost entirely on generating evaluations of local interventions.

### Recruitment

In each of the selected requests from the AskFuse scheme a lead PHP was identified and invited for interview by the AskFuse Research Manager by email. Once the lead PHP had been interviewed by the research team, the main Fuse researcher supporting the PHP’s request was also approached for interview. For the PHPES scheme a lead SPHR researcher was identified first for each of the applications that had been successful in applying for funding in its first two years of operation. The lead researcher was initially approached by the SPHR’s Deputy Director for permission to be contacted by the research team. Once the SPHR researcher had given permission and was interviewed, the main PHP for that application was approached. For unsuccessful PHPES applications, only the lead PHP was interviewed as they received no funded support from SPHR researchers (although all applicants were offered advice and referrals to other funding sources and support organisations). From the 20 sample requests, 11 PHPs and seven researchers were interviewed, of which four PHPs interviews related to unsuccessful applications or support requests (17 one-to-one via telephone, one face-to-face in the workplace), as indicated in Table 1.

### Data collection and analysis

Interview topic guides were developed and piloted in line with the research questions, and focussed on the barriers and facilitators to approaching researchers and engaging with research and evaluation, with key themes developed from existing theoretical and empirical research on knowledge exchange [2, 7, 8]. Interviews lasted between 20 and 90 min (average 40 min). All interviews were conducted by a female interviewer with five years of experience in public health research (LF).

Interviews were digitally recorded and transcribed verbatim. Participants were offered to review their transcript when completed; no participant opted to correct their transcript. The interviewer also kept field notes in a personal diary to reflect on her interview experiences and findings. Transcriptions were coded in NVivo using thematic framework analysis [11]. Initial coding was performed by the interviewer and each code was validated independently by three other research team members. Where coding discrepancies occurred, these were discussed in team meetings to agree on adjustments and refinements of the coding framework.

### Data verification

To verify and refine the findings from the interviews, an interactive workshop was organised in January 2016 involving PHPs and researchers based in the North East of England. Approximately 30 participants from a range of sectors attended the workshop. After being presented with three case studies of existing successful research-practice collaborations in the region, participants were invited to reflect on their own experiences and discuss in groups what made the case studies successful, what barriers existed to effective working between PHPs and researchers and what could be done to remove barriers. Towards the end of the workshop the research team presented findings from the interview study and related them to the group discussions. Barriers and solutions identified were noted, as were detailed notes of the group discussions. The notes were combined into a detailed report of the workshop. We report our methods and results in this paper according to the consolidated criteria for reporting qualitative research (COREQ) [12].

## Results

Firstly, we highlight the barriers and facilitators identified by our participants for engagement between PHPs and researchers. We compare these to the known barriers to collaboration from the literature to establish whether the two responsive research schemes help PHPs and researchers to overcome barriers. The comparison is followed by an in-depth exploration of additional issues identified by participants using both schemes, including recommendations for improving engagement by addressing the wider cultural and system issues that hamper evidence use and better communication.

### Barriers to engaging with research

Although a wide range of barriers to working with researchers was suggested, those mentioned most, which does not necessarily imply they are the most important, related to: the different timescales involved, where PHPs wanted a quick result and researchers could not deliver on this; the financial cost of involving researchers in a research project; and the difficulty in finding the most appropriate researcher with whom to work. These themes resonated with existing literature [2, 7, 8].

### Disconnected timescales

Often, where a local problem was identified by a PHP and their service, a solution needed to be produced within a short timescale, with researchers usually having insufficient flexibility to deliver to this timescale.

*So eventually we kind of developed a project but it took a couple of years to actually develop the project with [name] University and it’s in the [name] PhD so that’s three years minimum. So we’re talking five years. So this is at the same time, at the same meeting, the Chief Exec said “Yeah I want the strategy written in a month” [laughing]. So, you know, imperatives are very, very, very different* (public health professional/user, PHPES).

Public health professionals also suggested problems with competing sources of evidence and questions around what evidence it is feasible to generate within the required timescales and what type of research is appropriate within this timescale.

*…they’re keen that the evaluation we’re doing will show if the … intervention is having an effect or not on [*identified problem; anonymised*], when, actually, it’s not really at the point of being able to produce that kind of evidence. We’re going into it thinking of it as a feasibility study, and it’s almost really turning into a kind of qualitative study about how it’s being delivered and how the agencies are accessing the training and that kind of thing. I suppose, from our point of view, it’s at an earlier stage, really. It’s not at a point where we’re able to reliably collect evidence [of effectiveness]* (researcher, PHPES).

The quote not only illustrates a mismatch in timelines between research completion and input for decision making but also an issue about PHP understanding of what types of evidence can be extracted from different types of research, or from research at different stages of completion. In essence, the mismatch is a communication issue between the researchers and PHPs involved in the project, which requires attention in the early stages of proposal development.

### Costs of research

Participants also commented that rigorous research is expensive and the cost is often unaffordable by local public health teams.

*I think, particularly now, there is a lack of money. You might argue that that means that people want to show that things are actually working, but they just do quick and dirty ways of what they think, with a wee bit of feedback here and there that says, ‘This is working’* (researcher, AskFuse).

We would not wish to suggest that research conducted by local public health teams is ‘quick and dirty’. However, the quote does appear to reflect prejudice about the potential quality of research conducted by local public health team. What this quote illustrates is that resource-constrained LAs, as is the case in England presently [8], tend to commission small budget research that might not be as rigorous or of a scale as researchers would like to undertake. In response, researchers have tried to help by working with PHPs to seek funding from national funding organisations for rigorous research. However, researchers commented on the mismatch between local needs and funders’ priorities. For example, national funders may prioritise research designs that are not suitable for applied, local evaluations. Moreover, problems caused by the mismatch in priorities were exacerbated by the extremely lengthy timescales for application, peer review and contractual arrangements associated with external funding sources, with researchers usually unable to secure funding for flexible research designs that allow timely local evaluations.

*You go to somewhere like NIHR [National Institute for Health Research; a national research funder] and they say, “No, it’s not properly controlled.” It’s like, “Well, how on earth would you ever properly control this?”… I think the funding issue is a big one, yes* (researcher, AskFuse).

### Finding the appropriate people to work with

Public health professionals discussed their difficulties in finding the appropriate researcher with whom to work:

*So, again, you might also find that you’re passed around a number of departments in terms of what particular interests… Because I think, at the moment, as I say, it is quite hard to work out who to engage with, or how best to go about that. I’ve contacted academic institutions before and I’ve still not had a response, but that’s understandable, too. But, clearly, there needs to be a better way of doing it than that* (public health professional/user, PHPES).

Researchers also experienced difficulties in contacting appropriate PHPs, particularly since the most recent restructuring of public health services:

*But I think the big difference for me is some of those links. So I know a lot of people have moved to the local authority but that’s been quite a bit of change, and some of the people that I’ve worked with previously… The various commissioners and things have disappeared or changed. So that’s been a bit of an issue* (researcher, AskFuse).

### Understanding the evidence base

Other reasons that were suggested by PHPs for lack of engagement were related to lack of local relevance of national research findings, with research studies “*being too far removed from what is happening on the ground*” (PHP, PHPES), and inconsistencies between existing research findings. Contested evidence was also noted as a reason why research findings were not always valued.

*For me personally a frustrating thing is when a piece of research comes out, or a study comes out, and says one thing and then another study comes out, a couple of months later, and says completely the opposite. …it can be quite frustrating at times, when you do get a lot of conflicting advice and evidence around certain issues* (public health professional/user, AskFuse).

### How do responsive research services address barriers to engaging with research?

Through working with responsive research services, participants commented that they have been able to overcome some of the known barriers, particularly when trying to find the right researchers with whom to engage.

*I think AskFuse makes it a lot simpler and creates that relationship so that, you know, there is a really accessible way to academics and it starts to develop a relationship which actually makes practitioners realise well, actually, they are co-operative partners. It’s not a completely different sector. And I would imagine, being involved with AskFuse, it will open the door to collaboration* (public health professional/user, AskFuse).

Funding schemes such as PHPES further help to secure funding for local evaluations that is not available anywhere else. Both schemes enable PHPs to access useful evidence of a variety of types, such as qualitative findings on acceptability and feasibility of local interventions, and identification of mechanisms that enable successful implementation. Cost-effectiveness analyses also provided commissioners with input for the business cases they needed to make for sustaining funding of interventions. However, responsive research services did not always make the process easier or result in a match with suitable researchers.

*When I went through with [name] he did look through your database of potential available researchers and I think no one had the capacity at that point in time or probably the expertise* (public health professional/user, AskFuse).

In the experience of one researcher, having to communicate via a portal can also be a barrier:

*Because I actually found the process quite difficult. So often, you know an enquiry would come in but I need to know, this that or the other. But then having to say, “[AskFuse Research Manager], I need to know this that or the other”, and sometimes you kind of just want to say, “Can I just speak to this person, can I just ring them up?”... Or they wanted to deal with [AskFuse Research Manager], or [AskFuse Research Manager] wanted to deal with it or whatever. So sometimes I felt like the person in the middle was a bit of a barrier* (researcher, AskFuse).

Moreover, however much the services eased the building of partnerships, other issues, like the difficulties in aligning timelines, remained, with research processes, particularly around ethics procedures, taking more time than anticipated by PHPs.

Non-users of the services did not significantly differ in their views from the users interviewed and mentioned similar barriers and facilitators for engagement with researchers. They also valued research but had not yet engaged with responsive services for several reasons, such as a lack of time and resources for engagement, or having only unfocused research questions that they thought were not suitable for submission at the current stage. Other non-users deliberately chose to go elsewhere, often because they had already developed personal relationships with individual local researchers in the past.

Some of the PHPs had initially contacted one of the services but had not been successful in securing funding or the right researcher’s expertise and therefore did not engage further with the schemes. However, they still valued the support they had received, as it had helped them to focus their research ideas and to develop stronger proposals for future research. As a result, some of them had been able to secure internal funding.

### Deeper rooted issues

Participants highlighted deeper rooted issues at a cultural and system level that required additional support and change.

#### What evidence is valued?

Overall, there was strong consensus that PHPs and researchers did not always mean the same thing by evidence and that the type of evidence produced by researchers was not always the type required in practice, despite opportunities to access a wider variety of evidence types through the responsive research schemes, which were perceived to be more useful.

*It’s, you know, evidence is a whole range of different things and the views and the knowledge of local politicians for me is part of that evidence. So when we’re trying to develop local strategy that’s an important part, the views and the knowledge and expertise around residents and other parts of it, so yeah, I think possibly there is a bit of a gap between that, because locally we would see that whole range of the evidence if you like* (public health professional/user, PHPES).

The type of evidence favoured by PHPs tended to be in-house research or intelligence that had been produced locally, or in a similar area elsewhere. Researchers were thought not to understand or appreciate the value of this type of localised evidence. Lack of appreciation for the value of in-house research relates to the barrier we discussed around cost of research. PHPs preferred to commission smaller low-cost research projects, which might not be as rigorous as researchers would like, and valued the importance of political evidence, such as the perceptions of local constituents about an intervention, even if they did not use it. Since the move of public health to LAs, which marked a transition away from the consensual scientific view of evidence in the NHS, PHPs thought there was more reliance on such ‘soft’ or ‘grey’ evidence, which is information that accumulates from innovation in practice and that is informally published or not published at all [13]. Interestingly, the importance of soft evidence was also acknowledged by several researcher participants:

*Well, I think academics can be quite pedantic, which is what we’re trained to be in terms of NIHR-type standard and Cochrane standard evidence. Whereas, in the real world, the reality is that a newspaper article is taken as evidence, where a newspaper article is vaguely more credible than just what somebody has said, which is actually what policy may well be based on* (researcher, AskFuse).

#### Differences in structures

Some respondents referred to structural barriers resulting from researchers and PHPs speaking different languages. Structural barriers related to different priorities and expectations around evidence and research between PHPs and researchers. It often seemed that researchers wanted to answer a different question to that of the PHPs and to produce solutions that were not necessarily practical in the context in which they were required. Researchers were seen by PHPs to value different types of evidence from PHPs, even if they understood the need of PHPs for other types of evidence. The variation in priorities and expectations were linked by the participants to different incentive structures between research and practice worlds.

*I think there is always this conflict here, because in our side of the university there is always, we always have to think about REF [*Research Excellence Framework; a national assessment of the UK Universities’ research performance*], about publications, about grants – they are not able to offer to us a research design that would be of value for us to publish because - that wasn’t the main question. If I just took the results that they give to us and try to write a paper, I’m not sure I would be able to get it to a journal with high impact factor* (researcher, AskFuse).

Other participants framed the differences in priorities in terms of a lack of common ground, particularly since the move of public health to LAs:

*I think the challenge is being in an environment where you get the opportunity to understand what academia is doing and for them to understand what we’re doing. Certainly, since we’ve moved to local authority, we’ve lost some of that ability to do that. I was much more engaged in academia when I was in the NHS public health, than I am in local authority public health* (public health professional/non-user, AskFuse).

### Overcoming structural barriers

When asked to suggest ways that researchers and PHPs could work better together, similar suggestions were proposed by both PHPs and researchers. Suggestions focused on increasing awareness of each other’ structures and procedures, and the challenges faced by each profession within their respective organisational environments.

*I suppose it’s just greater awareness of the structures that public health operates in now, because it is very different since we left the NHS and we’re now part of LA. [..] It has to be done through a [*particular*] process. So I suppose closer relationships between academia and public health, on a day to day basis, on a practical day to day level, will hopefully enhance the knowledge of academia in terms of the procedures that we have to operate around* (public health professional/user, AskFuse).

There was a considerable agreement between PHPs and researchers that it would be useful to work together from the early developmental stages of a research project. However, it was also recognised that research was often considered as an afterthought in practice, with researcher input coming too late. With increasing austerity measures in LA, increasing the level of engagement was felt to be a major challenge.

#### Develop systems for collaboration

Embedding researchers in practice was suggested as one way to help bridge the gap between research and practice. One respondent anticipated such an arrangement that had been brokered through Fuse.

*I think the fact that we’re going to have a researcher in with the team. I think it’s things like that where, rather than just come in and support on a particular piece of work, actually being there as part of a public health team, seeing the work that goes on, and really getting involved in a piece of work and experiencing some of the pressures, and experiencing some of the issues, and the barriers, and the problems and the challenges, we face as PHPs in the current climate, and in the current organisations that we work in. Being there on a regular basis and seeing that, I think, adds an additional dimension to the research that will take place* (public health professional/user, AskFuse).

In addition, other ways to develop relationships were suggested, including sessional commitments.

*[…] it would be good to be able to develop a model where […] every Thursday afternoon, an academic or researcher in such and such an area always spends that time working with an organisation on a pro-bono basis or something. I don’t know. In return for that, the organisation will feed in intelligence that they’re receiving to influence and shape the work that academics might do in terms of their research priorities or that kind of thing, I guess* (researcher, AskFuse).

Having a forum to informally discuss issues and bring people together was also suggested. Often there are research focussed meetings on specific topics where sometimes PHPs can attend but it was thought that less prescriptive sessions to discuss ideas, introduce people, build partnerships and develop research ideas could work better.

*Quite often, when I see events, it’s because it’s looking at a particular research area that is being conducted rather than providing just the space for discussion around, “Are people interested in working together?” and looking at that kind of thing* (researcher, AskFuse).

### Verifying findings and identifying solutions: Interactive workshop with PHPs and researchers in the north east

To verify and refine the findings from the interviews, an interactive workshop was organised in January 2016 involving PHPs and researchers based in the North East of England. Participants attended the workshop confirmed the veracity of the barriers identified in the interviews and provides suggestions for potential solutions. They reinforced the need for understanding each other’s systems. Mismatches in timescales between researchers and PHPs were acknowledged but both professions urged each other to show persistence in developing initial small-scale projects into larger and longer-term collaborative studies. Securing quick wins, such as collecting base-line data or undertaking qualitative research to inform intervention development, was felt to be important for building relationships and confidence between partners. Encouraging earlier-career staff, in both research and PHP roles, to take on collaborative opportunities was seen as particularly fruitful – either informally, or as part of and helping to achieve formal learning outcomes.

The co-writing of case studies of emerging, novel practice was identified as a possible route for achieving several outputs and building relationships. Alongside a quick win for both parties, it was felt that they might provide opportunities for PHPs to develop skills in research publication, as well as addressing researchers’ needs for outputs and providing researchers with greater insight into everyday public health practice. Using reporting checklists for co-writing case studies, such as the TiDIER framework [14], would provide a standardised format that could support shared learning between PHPs in different localities. Open-access publications in journals for PHPs were felt to be particularly important. Funding for collaborative projects was seen as a particular problem. National funding organisations were encouraged to develop flexible funding models that encouraged novel, and potentially high-risk, practice-based research; for instance, natural experiment evaluations and qualitative case studies, and to develop mechanisms to fund intervention costs within evaluative research.

## Discussion

This study contributes to the knowledge exchange literature by examining collaborations between researchers and PHPs engaged in responsive research schemes that have been developed to tackle existing barriers to evidence generation and exchange.

### Principal findings

Participants recognised the importance and value of research evidence for informing practice and decision making. However, PHPs identified three main barriers that prevented them from engaging effectively with researchers: 1) differences in timescales; 2) limited budgets; and 3) difficulties in finding the most appropriate researchers to meet their needs. The two responsive research schemes were able to address some of these barriers, particularly by matchmaking researchers with PHPs and by providing a context in which exploratory conversations about mutual interests could flourish. The schemes gave access to evidence syntheses and could support focused queries or shaping of projects but other barriers remained, such as mismatched timescales and the need for more research resources.

Additionally, participants highlighted deeper rooted cultural and system level issues. Thus, PHPs felt that researchers did not fully understand the structures and environments in which they worked and their challenges. In particular, the recent move of public health services to LA was associated with a less rigorously evidence-based culture and a more rigid tendering process, while fewer financial resources were available in a climate of austerity [15]. Different types of evidence were felt to be necessary to provide the financial justification for proceeding with some work.

Researchers similarly felt PHPs did not understand the environment in which they worked. Particular issues identified were the high costs of fully economically costed research, the rigorous demands of research governance and ethics procedures and associated demands on time, the constraints of project-based funding models leaving little scope for exploratory work, and institutional pressures to publish in high-impact journals. Despite the growing salience of the ‘impact’ agenda throughout academia [16], structural barriers remain: lack of local resources and limited institutional incentives to engage in collaborative applied research are ongoing challenges for researchers.

Participants’ key proposed solutions for overcoming these structural issues focused on the exploration of opportunities for researchers to spend time in policy or practice settings, the creation of open forums and opportunities for exchange, changes in the priorities of research funders, identifying funding for intervention costs in the context of research studies and provision of free access to journal publications for public health departments.

### Strengths and limitations

The qualitative research design, sampling of both users and non-users of responsive research schemes and the researchers who support them, together with a solution-focused workshop, add depth and understanding to the issues raised by PHPs and researchers.

The response from participants in the PHPES scheme was more limited than for AskFuse, with fewer non-successful applicants from the PHPES scheme participating in interviews, and no researchers being interviewed that supported non-successful applications, which potentially limited generalisability. Despite these limitations, our analysis of the interview data reached thematic saturation and our findings are in line with previous studies [2, 8, 9] which suggests a degree of validity of the results.

The principal interviewer was a researcher and based in Fuse, as funding for the study was awarded to Fuse, which may have affected the responses of participants. However, the interviewer had no prior involvement with AskFuse or PHPES. It was partly in response to potential bias that we also held the interactive workshop, which offered opportunities for in-depth discussion of the issues and allowed PHPs and researchers to be challenging. The workshop also allowed the emergence of consensus on some of the issues raised in interviews, as well as discussion that was solution-focussed.

Both services studied were set within an English context and therefore different issues might apply in other countries with different governance and health systems. However, the literature suggests the ubiquity of these concerns [8]. Austerity measures and subsequent financial pressures in public heath are experienced across the globe and will continue to drive a need for spending wisely on what works, based not only on the most scientifically rigorous but also the most practically useful evidence for PHPs to utilise in informing policy and practice.

### Implications for research, policy and practice

The solutions identified in our study by both PHPs and researchers have the potential to go some way towards overcoming barriers, but most are untested. For instance, embedded research approaches are currently popular but only limited evaluations of such schemes and mainly in clinical settings [17] have been undertaken. Moreover, structural barriers are difficult to address through response research schemes alone. In the UK, public health budgets seem likely to remain restricted for the foreseeable future, which will hamper the commissioning of even small-scale research. Whilst, there may be much to learn from other jurisdictions where the health systems and governance arrangements may differ, some of the underlying issues that determine translation may be similar [18].

It will also take time to shift the priorities of research funders towards research that is relevant to ‘end users’ and that will increase institutional incentives for researchers to engage through collaborative research with end users. Shifting priorities might be achieved through extensive consultation about service needs when funder-led research agendas are being set [19] or through acceptance of a wider and flexible range of research designs, including evaluations of natural experiments [20], which may have more appeal to local users because of their greater ability to consider local context [21].

Increasing emphasis on impact in quality assessments of research, such as the UK Research Excellence Framework 2021 [16] might help to further encourage engagement between researcher and PHPs, provided that these frameworks allows for flexible approaches to measuring impact which take account of the barriers identified in this study. For example, Impact can occur without the publication of high quality peer reviewed publications, and can be based on evidence that is more broadly defined and set alongside political priorities and local responsiveness in Local Government [5].

At the same time as the research world changes its processes to better address PHPs’ needs, it might be possible to support the development of research capacity in practice settings. Increasing research capacity might be achieved through both locally tailored training courses in research skills and through supportive collaborations which encourage PHPs, even where engaged in small-scale local evaluations, to use standard evaluation frameworks [22] and standard reporting processes. Using standards could encourage systematic approaches and rigour in small scale research and facilitate the development of databases of local public health practice and research activity that could act as national resources.

Overall, our study highlights the need for stronger collaborations between researchers and PHPs and a high level of enthusiasm for developing novel solutions to the problems identified.

## Conclusions

Responsive research schemes appear to overcome some of the challenges to collaboration between researchers and PHPs, but other barriers remain. Remaining barriers primarily revolve around system differences in priorities as well as structural barriers, such as incentive structures, that prevent informal and frequent meetings between researchers and PHPs. Structural barriers result in a persistent mismatch in appreciation of the purposes, potential and impact of research among PHPs and researchers.

A range of measures will be necessary to ensure a research system supportive of public health practice. In particular, the enhancement of dialogue with researchers about what matters to PHPs, utilising more dynamic and fluid approaches to knowledge exchange [23] should be a key driver of research agendas if the end products are going to have relevance to policy and practice and change cultures and perceptions on knowledge exchange within universities. Research funding schemes need to be alert to PHPs needs and develop in ways which are reflective of the new public health landscape, without necessarily compromising scientific integrity.

Above all, we argue that an increased mutual awareness of the structures and challenges within which both PHPs and researchers are working is required as a first step towards breaking down remaining barriers. The need for increased mutual awareness extends beyond the UK context, as the literature suggest that the barriers for collaboration between researchers and PHP are not unique to the UK [8]. There is a global interest in exploring collaborative models such as embedding researchers in practice settings to enable this [24]. As these models of collaboration and exchange are mostly untested, research will be needed to develop and evaluate them. Our study did not intend to solve identified problems for collaborative research; future research could explore and test innovative strategies for addressing remaining structural and cultural barriers for collaboration.

^1^run by the UK National Institute for Health Research School for Public Health Research.

^2^run by Fuse, a UK Clinical Research Collaborations funded consortium of public health researchers in the North East of England.

## Abbreviations

LA: Local authorities
NIHR SPHR: National Institute for Health Research School for Public Health Research
PHP: Public health professionals
PHPES: Public health practitioner evaluation scheme
UK CRC: United Kingdom clinical research collaborations
